# Reactive Oxygen Species and Antioxidants in Postharvest Vegetables and Fruits

**DOI:** 10.1155/2020/8817778

**Published:** 2020-12-10

**Authors:** Karlia Meitha, Yonadita Pramesti, Sony Suhandono

**Affiliations:** School of Life Sciences and Technology, Institut Teknologi Bandung, Jl. Ganesha No. 10, Bandung 40132, Indonesia

## Abstract

Reducing oxidative species to non- or less-reactive matter is the principal function of an antioxidant. Plant-based food is the main external source of antioxidants that helps protect our cells from oxidative damage. During postharvest storage and distribution, fruits and vegetables often increase ROS production that is quenched by depleting their antioxidant pools to protect their cells, which may leave none for humans. ROS are molecules produced from oxygen metabolism; some of the most widely analyzed ROS in plants are singlet oxygen, superoxide, hydrogen peroxide, and hydroxyl radicals. ROS concentration and lifetime are determined by the availability and composition of the antioxidant system that includes enzymatic components such as SOD, CAT, and APX and nonenzymatic components such as vitamins, polyphenols, and carotenoid. Depending on its concentration in the cell, ROS can either be harmful or beneficial. At high concentrations, ROS can damage various kinds of biomolecules such as lipids, proteins, DNA, and RNA, whereas at low or moderate concentrations, ROS can act as second messengers in the intracellular signaling cascade that mediates various plant responses. Novel postharvest methods are sought to maintain fruit and vegetable quality, including minimizing ROS while preserving their antioxidant content.

## 1. Introduction

Fresh fruits and vegetables are major external sources of antioxidants for human, which consist of mostly polyphenols, flavonoids, and vitamins [1]. Routine consumption of natural antioxidant is proposed to reduce the risks of lifestyle-related illness such as diabetes, obesity, and cardiovascular disease [2]. However, antioxidant content decreases once fruits or vegetables are harvested and even more rapidly in poor postharvest storage or distribution. The generation of reactive oxygen species (ROS) during these conditions depletes antioxidants through a plethora of reduction-oxidation reactions.

ROS are reactive molecules produced from oxygen metabolism. Mitochondria, chloroplasts, and peroxisomes are the main organelles generating ROS in plant cells. Depending on their concentration in the cell, ROS can be either harmful or beneficial. At a high concentration, ROS can damage various kinds of biomolecules, whereas at low or moderate concentrations, ROS can act as second messengers in intracellular signaling [3]. ROS signaling is crucial during germination [4] and through to perception of bud bursting in perennial plants [5]. However, in postharvest commodities where growth or morphological change is no longer desired, the presence of ROS without antioxidant is avoided.

Postharvest management of fruits and vegetables, in the context of balancing ROS and antioxidants, is not simple. Some methods are able to augment antioxidant production, but often at the price of increasing ROS beforehand [6–9]. Increased ROS in postharvest commodities is beneficial to impede pathogen infection, which is then counterbalanced by antioxidant upsurge to protect plant cellular machineries. Promising methods include the use of hormones [10], antagonistic yeasts [11], coating [12], and genetic engineering [13] among others.

**Figure 1:**
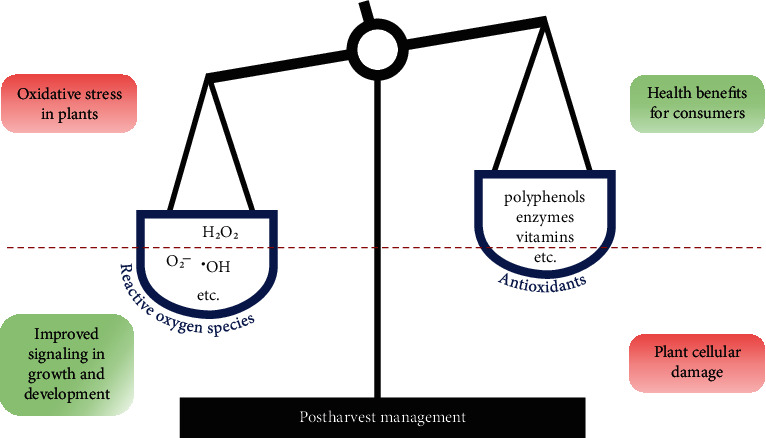
Postharvest management, balancing ROS and antioxidant content to maintain quality and deliver the most benefits to the consumer. The broken red line illustrates the fragile balance between ROS and antioxidant in plants. A moderate level of ROS is often beneficial to signal various crucial steps during growth and development, including ripening and increased resistance in a postharvest condition. However, elevated ROS concentration that is not coupled with high antioxidant capacity often poses great risk to the plant tissues. Hence, a suitable method of postharvest would be one that is able to limit ROS production while maintaining high antioxidant content.

This review will discuss the types of ROS that are most frequently analyzed in plants, including their damaging behavior, production and degradation mechanism, and role in signaling related to postharvest management (Figure 1). Major antioxidants in fruits and vegetables will also be examined, including the enzymatic (sodium dismutase, catalase, guaiacol peroxidase, ascorbate peroxidase, glutathione peroxidase, and glutathione reductase) and nonenzymatic antioxidants (vitamins, flavonoids, and carotenoids) [1]. Plant perception to both external (light, oxygen level, pressure, wounding, microbial infection, etc.) and internal (senescence, ripening, etc.) cues involves a burst of ROS content. In a postharvest situation, multiple cues are often received at the same time, which triggers a critically high level of ROS.

## 2. Types, Production, and Degradation of ROS in Plant Cells

The most analyzed ROS in plant signaling are singlet oxygen, superoxide, hydrogen peroxide, and hydroxyl radicals [3]. Reduction of oxygen (O_2_) produces superoxide (O_2_^−^), which is then dismutated into hydrogen peroxide (H_2_O_2_). Hydrogen peroxide then can be reduced partially to the hydroxyl radical (∗OH) or fully to water (H_2_O) [14]. It is worth mentioning that about 1% of O_2_ consumed by plants is converted to produce ROS [15].

Singlet oxygen is a by-product of photosynthesis in plants, in which due to chlorophyll change with the help of light converts triplet oxygen (^3^O_2_) into singlet oxygen. Moreover, this molecule is highly reactive with organic compounds that contain double bond and it affects negatively the efficiency of photosynthesis. Hence, plants exposed to excessive light can produce an abundance of singlet oxygen to the extent that it can eventually cause cell death. Several molecules, such as carotenoids and plastoquinone in chloroplasts, can counteract and thus balance the negative effects of singlet oxygen [16].

Hydrogen peroxide is also commonly produced in cells either under normal conditions or under stress conditions such as drought, cold, radiation, and UV. The main sources of hydrogen peroxide production in cells are the chloroplast and mitochondrial electron transport chain as well as photorespiration [3]. Hydrogen peroxide can pass through the membrane such that it moves through the cell walls and causes oxidative damage far from its place of origin. Because of these capabilities, hydrogen peroxide is now widely studied as a signaling molecule that plays a role in the regulation of biological processes [17]. In high concentrations, hydrogen peroxide can oxidize cystine or methionine residues and inactivated enzymes by oxidizing the thiol groups of these enzymes. It can also oxidize protein kinase, phosphatase, and transcription factors that contain thiolate residues [3].

The most reactive ROS is the hydroxyl radical (∗OH). This species has a pair of unpaired electrons that can react with oxygen in the triplet ground state. Hydroxyl radicals can also interact with all biological molecules and cause cell damage in lipids (peroxidation), proteins, and membranes. Excessive production of hydroxyl radicals can cause cell death because cells do not have enzymes to eliminate the radicals [14].

In general, ROS can be produced in almost all compartments of a plant cell. Most cell compartments have antioxidant systems that act as redox buffers. However, ROS concentration and lifetime are determined by the availability and composition of these antioxidant systems [18]. For example, the lifetime of hydroxyl radicals, which is the most reactive form of ROS, is estimated to be in the order of nanoseconds, while for singlet oxygen, it is in microseconds. The lifetime of hydrogen peroxide and superoxide is estimated to be in the order of milliseconds to one second, among the longest in ROS [19].

ROS in chloroplasts is related to the light reaction of photosynthesis. Singlet oxygen is produced as a by-product of photosynthesis in plants. Photosensitizers, such as chlorophyll, convert triplet oxygen (^3^O_2_) into singlet oxygen with the help of light [20]. In chloroplasts, there are no enzymes that break down ROS directly, and therefore, ROS is broken down with the help of other molecules such as carotenoids and tocopherol [21].

The superoxide is also produced in chloroplasts as a result of oxygen reduction in photosystem. This ROS molecule will be dismutated into hydrogen peroxide by sodium dismutase in chloroplasts, in the form of iron-SODs (FeSODs) and copper/zinc-SODs (Cu/ZnSODs). Hydrogen peroxide is then detoxified by ascorbate peroxidase (APX), glutathione peroxidase-like enzymes (GPXLs), and peroxiredoxin [15].

The main source of ROS in mitochondria is the complexes bound to the inner membrane, which is involved in mitochondrial electron transfer chain (mETC) [22]. ROS in the intermembrane space (IMS) is broken down by components from the cytoplasm because of the permeability of the mitochondrial outer membrane, while the antioxidant system in the matrix is carried out by the manganese-SOD enzyme, wherein the hydrogen peroxide formed is broken down by ascorbate peroxidase and peroxiredoxin enzymes [23].

ROS in peroxisomes is formed from the oxygenation process of glycolic that produces glycoxylate and hydrogen peroxide as by-products. Glycolic is metabolized and transported to peroxisome from 2-phosphoglycate in chloroplasts. Prior to that, 2-phosphoglycate is produced from the oxygenation of ribulose-1,5-biphosphate by RuBisCO in chloroplasts. The antioxidant system in peroxisomes is carried out mainly by the catalase enzyme [24].

Apoplasts play a role in the exchange of nutrients and signals between plant cells and the surrounding environment. In many cases, plants respond to an environmental stimulus because of the accumulation of ROS in this compartment [20]. ROS accumulation in apoplasts is a consequence of apoplastic peroxidase, polyamine oxidase (PAO), and NADPH oxidase activities. PAOs catalyze the catabolism of spermine and polyamine spermine with hydrogen peroxide as by-products, whereas NADPH oxidase transfers electrons from NADPH to O_2_ and produces superoxide. The resulting superoxide is dismutated to hydrogen peroxide either spontaneously or by superoxide dismutase [16].

During the postharvest condition, leafy vegetables and other green tissues remain photosynthetically active. This is problematic as removing the light accelerates chlorophyll degradation, as reviewed by Zhu et al. [25]. Further, darkness also increases ROS production in mitochondria, especially in nonphotosynthetic tissues [26] such as the flesh of fruits. This yellowing of fresh produce with reduced nutrient content during postharvest storage leads to major losses in the agriculture industry.

## 3. ROS: Friend or Foe

In past decades, ROS were often considered dangerous molecules due to their destructive nature towards various molecules in cells when present at a high concentration. Some of these harmful effects include the destruction of DNA and RNA, oxidation of lipid and amino acids, and deactivation of enzymes by oxidizing the cofactors of these enzymes [4], which could eventually cause cell death. This assumption has changed, however, and ROS are currently considered molecules that play a role in signaling. The higher rate of ROS production influenced by the environment causes a change in redox balance in the compartment to a more oxidized state, which can then be captured as a signal by the system in the compartment to regulate gene expression. Thus, the state returns to a balanced one [16].

In low or moderate concentrations, ROS can act as second messengers in the intracellular signaling cascade that mediates various plant responses, including stomatal closure, apoptosis, gravitropism, and the acquisition of tolerance to biotic and abiotic stress [3]. Plants can recognize ROS signals and direct and interpret them into the proper cell response with the help of redox-sensitive proteins. ROS can also modulate the activity of various components in signaling, such as protein phosphatase, protein kinase, and transcription factors [4]. One example of the role of ROS as a second messenger is in the closing of stomata. Here, hydrogen peroxide-induced ABA acts as an important signal in mediating stomatal closure to reduce water loss through the activation of channels in the plasma membrane [27].

ROS also play a role in sending cell differentiation signals in various parts of the plant. In dormant seeds, plant embryos and their surrounding endosperm show a low level of metabolic activity. But after imbibition and during germination, metabolism and ROS content increase rapidly. Hence, increased ROS production acts as a positive signal for germination [28]. Furthermore, this molecule also plays a role in the development of plant organs. Foreman et al. [29] suggested that ROS homeostasis could limit the growth of main roots, trigger the emergence of lateral roots, and increase root hair growth. Meitha et al. [5] also mentioned that ROS also take part in initiating the process of bud burst in grapevines. During a bud burst of a shoot, the meristem undergoes a transition from hypoxia to increasing pO_2_, followed by accumulation of ROS in the developing cambium and vascular tissue, colocalizing with lignified cellulose association with the provascular tissue. Ogawa et al. [30] showed that there was strong colocalization between lignin and superoxide in the vascular tissue of spinach.

ROS are also instrumental in the development of flowers, especially in the development of petals, pollen tubes, and gametophytes [31]. Further, ROS are also responsible for fruit development and ripening. Huan et al. [32] showed that in the middle stage of peach development, there is a high accumulation of superoxide and hydrogen peroxide which is closely followed by the rapid development of the fruit. Thus, it is also suggested that increased ROS production acts as a positive signal for development and maturation. ROS also play a role in the development of maize leaves as Rodríguez et al. [33] demonstrated through a high accumulation of hydrogen peroxide in the elongation zone of maize leaves. Observations using electron micrographs show that hydrogen peroxide is mostly accumulated in the apoplast. Hence, ROS play a crucial role, mostly in signaling, during plant development and ripening in fruits. However, in postharvest fresh fruits and vegetables, physical and physiological changes are usually avoided.

## 4. ROS-Related Postharvest Damage

The declining quality of fresh vegetable and fruit commodities due to poor postharvest management is a major problem in Asia. According to Blakeney [34], the Food and Agriculture Organization of the U.N. predicts that about 120 kg of food at preconsumption stages are wasted or lost per year. Harvested vegetables and fruits are vulnerable to changes in structure, nutrition, and biochemistry, which could accelerate decay. Furthermore, microorganisms and loss of water inevitably reduce quality of the fresh commodities [35].

In general, spoilage in postharvest vegetables occurs due to the loss of water content in vegetables and also microbial activity. Loss of water content in vegetables can cause cell damage and plasmolysis that leads to injury [35]. The injured tissue induces a higher respiration rate that triggers the rapid occurrence of tissue damage. In addition, tissue injury also increases the production of ROS that leads to subsequent molecular and physiological damage [36].

During postharvest storage, the process of transpiration in fruits and vegetables continues, which exacerbates water loss in cells. Water loss can also occur due to external injuries such as careless handling that causes bruising and skin damage. In addition, the ongoing respiration process uses stored sugar and stops when the stock runs out, leading to aging. Poor ventilation can also cause a lot of carbon dioxide to accumulate, and high levels of carbon dioxide quickly ruin produce. The microbial activity that remains during respiration also promotes higher ROS content during postharvest storage [37].

Spoilage in postharvest vegetables can also occur due to diseases caused by microbial activity that usually occurs in the field before being harvested. Harvesting or handling injuries can also cause microbes to enter the tissues of vegetables or fruits and cause damage during storage. Further, ethylene produced during storage accelerates fruit maturation which can further lead to storage losses. Ethylene production also increases when the fruit is injured and accelerates the decay, thus increasing ROS levels [35]. In this case, climacteric fruits are more prone to losses related to ethylene and possess a shorter shelf life.

High ROS levels induce lipid peroxidation activity in cells. Lipid peroxidation is known to exacerbate oxidative stress through the production of radicals from the peroxidation process which then react with the lipids themselves. Radicals produced, in addition to damaging lipids, also damage proteins and DNA [38]. Lipid peroxidase concentration is now widely used as an indicator of damage caused by ROS under stress conditions [3]. The two sites on the phospholipid molecules that are targeted by ROS are the unsaturated (double) bond between two carbon atoms and the ester bond between glycerol and fatty acids [39]. Unsaturated fatty acids present in membrane phospholipids are also sensitive to ROS attack. Only one molecule of hydroxyl radicals is needed to cause many unsaturated fatty acids to oxidize [38].

Proteins attacked by ROS can undergo several modifications either directly or indirectly. Direct modification occurs by modulation of protein activity through nitrosylation, carbonylation, formation of disulfate bonds, and glutathionylation. In contrast, indirect protein modification occurs by conjugation with products from fatty acid peroxidation [40]. ROS affect proteins in different ways, such as fragmentation of the peptide chain, change on the electrical charge, and modification of amino acids among other effects; the impacts often result in proteolysis [41]. Tissues damaged by oxidative stress will usually contain more concentrated carbonated protein concentrations which are then used as a marker of protein oxidation in these locations [42]. Each amino acid in the peptide has a different level of susceptibility to ROS attack with thiol and protein groups, which contain sulfur, having a very high level of susceptibility to ROS attack [43].

ROS is the main cause of DNA damage. The negative impact of ROS on DNA, among others, is that it causes deoxyribose oxidation, loss of nucleotides, modification of nitrogen bases, and cross-linking between DNA and protein [44]. DNA is the genetic material of cells, and damaged DNA can cause changes in the protein formed that eventually promote the formation of malfunctioning proteins. In addition, changes in the nucleotides in one chain can cause incompatibility with nucleotides in other chains, eventually causing mutations [45]. Oxidative attack on DNA generally occurs by the addition of ∗OH to DNA double bonds, whereas sugar damage occurs due to hydrogen extraction from deoxyribose. Hydroxyl radicals are also known to react with all of the purine bases and pyrimidines [3]. Hence, counteraction to these negative effects due to ROS accumulation in a postharvest condition is required.

## 5. Antioxidant System in Edible Plants

Antioxidants are found mainly in fresh vegetables and fruits and also in other foods such as nuts, meats, poultry, and fish. Consumption of vegetables and fruits is important to maintain a healthy body, especially when fighting against free radicals that damage cells and trigger chronic illnesses. As systematically reviewed by Swallah et al. [46], the antioxidant content of fruits, mainly polyphenols, has great potential to reduce the risk of lifestyle-related diseases. According to Giada [47], the top five fruits with the highest content of polyphenols are grape (*Vitis vinifera*), lemon (*Citrus limon*), kiwi (*Actinidia deliciosa*), lime (*Citrus aurantiifolia*), and black raspberry (*Rubus occidentalis*).

However, consumers are often unable to get this benefit because antioxidant content is heavily degraded during postharvest storage. According to Gil et al. [48], the storage process during postharvest reduced the content of antioxidants such as vitamin C and the scavenging activity of flavonoids in spinach. Del Caro et al. [49] suggested that the postharvest storage process can reduce the antioxidant content of ascorbic acid in citrus fruits. Furthermore, Hounsome et al. [50] showed that long-term storage significantly reduced the content of ascorbic, caffeic, syringic, and gallic acids, as well as flavonoids of white cabbage.

Plants have a complex defense system against oxidative stress. The defense system includes enzymatic and nonenzymatic components [51]. The enzymatic components include the superoxide dismutase (SOD), catalase (CAT), guaiacol peroxidase (G-POD), ascorbate peroxidase (APX), glutathione peroxidase (GPX), glutathione reductase (GR), and other enzymes. These enzymes work in different cell compartments and respond when cells experience oxidative stress [14].

Superoxide dismutase is an enzyme that plays a role in dismutating superoxide into oxygen and hydrogen peroxide. SOD activity is known to increase when plants experience environmental stress, such as drought and salinity. Thus, increased SOD activity often correlates with an increase in plant tolerance to environmental stress [3].

Another antioxidant enzyme is catalase, which is a tetrameric ubiquitous enzyme that contains heme. Catalase catalyzes the dismutation reaction of the two hydrogen peroxide molecules into water and oxygen [20]. Catalase has high specificity to hydrogen peroxide but has low activity against organic peroxide. The CAT enzyme has a lower affinity when compared to APX, but has a higher rate of turnover [4]. According to Willekens et al. [52], in tobacco plants, there are three kinds of CATs based on the profile of the tobacco gene expression. Class I CATs are expressed in photosynthetic tissue and regulated by light. Class II CATs are widely expressed in vascular tissue, whereas Class III CATs are widely expressed in seeds. Environmental stress on plants can cause CAT activity to increase or decrease, depending on the intensity and duration of stress as well as the type of environment itself.

The ascorbate-glutathione (AsA-GSH) cycle is a cycle consisting of reactions aimed at removing hydrogen peroxide. There are four enzymes involved in this cycle: APX, GR, monodehydroascorbate reductase (MDHAR), and dehydroascorbate reductase (DHAR). As reducing molecules, AsA and GSH are required to keep this cycle going [20]. The reaction catalyzed by APX is the transfer of electrons from AsA to hydrogen peroxide to produce monodehydroascorbate (MDHA) and water. MDHAR then catalyzes the regeneration reaction of AsA from MDHA using nicotinamide adenine dinucleotide phosphate (NADPH) as a reducing agent with a by-product of dehydroascorbate (DHA). Furthermore, DHA is reduced to AsA with the help of DHAR and GSH as electron donors. The last enzyme of this cycle is GR and plays a role in catalyzing the reduction reaction of glutathione to GSH with the help of NADPH [3].

Another type of antioxidant enzyme is GPX. GPX contains heme and functions in oxidizing aromatic donor electrons such as guaiacol and pyrogallol [20]. GPX is found in animals, plants, and microbes. Isoenzymes from GPX are found only in vacuoles, cell walls, and cytosols. The enzyme also plays a role in several biosynthetic processes such as cell wall lignification, IAA degradation, and ethylene biosynthesis. GPX is widely produced by cells when cells experience oxidative stress [53].

In plants, apart from enzymatic components, ROS are also broken down with nonenzymatic components such as AsA, GSH, *α*-tocopherol, carotenoids, flavonoids, and proline [3]. AsA and GSH work as antioxidants by donating electrons to ROS molecules. AsA also plays a role in signaling and modulation of gene expression related to enzyme activity. AsA and GSH act as substrates for certain peroxidases and are located in the chloroplast, mitochondria, nucleus, peroxisome, cytosol, vacuole, and endoplasmic reticulum [54]. As a lipophilic antioxidant, tocopherol can break down ROS and lipid radicals. For this reason, tocopherols play a major role in protecting cell membranes [16]. Among the four tocopherol isomers found in plants, *α*-tocopherol has the highest antioxidant capacity. As such, tocopherol plays a role in singlet oxygen degradation such that it can protect photosystem II and prevent lipid peroxidation propagation. *α*-Tocopherol levels in cells can change significantly in reaction to environmental stress [53].

Carotenoids, lipid-soluble compounds, act as antioxidants by protecting the cell's photosynthetic machinery. They eliminate the excess excitation energy through the xanthophyll cycle, thereby preventing the formation of oxygen singlets by reacting with excited chlorophyll molecules [55].

Polyphenols are one of the most important antioxidants in plants and are often found as flavonoids, gingerols, polyphenolic acid, and curcumin. They contain aromatic rings with -OH or -OCH_3_ substituents that play a role in antioxidant activity. Polyphenols can also damage ROS species and inhibit lipid peroxidation by binding to lipid alkoxyl radicals. Flavonoids are also known to be oxidized by peroxidase and play a role in H_2_O_2_ degradation [3].

Another phenolic compound that can act as an antioxidant is curcumin. Curcumin is the main component of *Curcuma longa* L. that works as an antioxidant by the electron transfer mechanism and also protects membranes from peroxidative damage. Curcumin includes many functional groups, including the *β*-keto group, carbon-carbon double bond, and phenyl rings [56]. Curcumin is degradable to ferulic acid and vanillin, two well-known antioxidants.

In addition to polyphenols, protein, vitamins, and minerals can also act as antioxidants. Proteins can inhibit lipid oxidation, one way of which is by using iron-binding variants. Protein also inactivates ROS, scavenges free radicals, and reduces hydroperoxides [57]. Vitamins and minerals are micronutrients needed in small amounts. One of the most well-studied vitamins with antioxidant properties is vitamin C or ascorbic acid. Vitamin C works as an antioxidant in the aqueous phase of the cell and circulatory system by donating high-energy electrons to neutralize free radicals. It also plays a role in regenerating the antioxidant form of vitamin E by reducing tocopheroxyl radicals. Other antioxidants such as albumin, hepatoglobin, and nonprotein antioxidants such as bilirubin and ubiquinol can also be found in plants [58].

## 6. Balancing Act of ROS and Antioxidant in Postharvest

There are several ways to slow decay in fruits and vegetables such as treatment with low temperatures [59], atmosphere control [60], hormones [10], and coating [12], which are summarized in Table 1. The cooling process can reduce the metabolic rate of fruits and vegetables [59]. According to Kan et al. [61], the cooling process in peaches can reduce the accumulation of ROS and reduce the level of membrane lipid peroxidation; thus, it can delay the senescence of peaches. Cold storage of the peach at 5°C prompted higher activity of antioxidant enzymes, such as succinic dehydrogenase (SDH) and cytochrome C oxidase (CCO).

Decay can also be prevented by atmospheric modification. The air composition normally consists of O_2_ (20%), CO_2_ (0.03%), and N_2_ (78.8%). Modification of air composition is done by reducing oxygen levels or increasing carbon dioxide content. The low oxygen content in the air and the increase in carbon dioxide content result in a decrease in the rate of the respiratory activity of vegetables and fruits [60]. Reduction in the rate of respiratory activity can then decrease production of ROS, because these species are produced mainly as the by-product of the respiration process. Therefore, the cooling process and modification of air composition can prevent damage caused by ROS and slow decay in fruits and vegetables. According to Song et al. [62], Modified Atmosphere Packaging (MAP) lowered the production rate of superoxide and hydrogen peroxide. It can also maintain high activities of superoxide dismutase (SOD), catalase (CAT), and ascorbate peroxidase (APX).

Environmentally friendly edible coating technology is currently being developed to overcome the problem of spoilage in postharvest vegetables, and one of the most promising options is chitosan coating. Chitosan is a by-product of the maritime industry, obtained from marine animal waste that is biodegradable and safe to the environment. It is able to form a barrier on the surface of vegetables or fruits with the effect of reducing respiration rate and loss of water to prevent spoilage [72], and it might decrease the production of ROS. Another characteristic of chitosan is the ability to increase antioxidant enzyme activity according to research on loquat fruit, strawberries, and bananas conducted by Adiletta et al.[59], Petriccione et al. [63], and Hosseini et al. [64], respectively.

An antagonistic effect of treating fruit with yeasts has also been shown to reduce ROS and increase antioxidant activity [11]. Harvested citrus fruits have been treated with coinoculation or codipping in *Pichia membranaefaciens* and pathogen fungi of *Penicillium italicum* and *Penicillium digitatum*. The results showed that both treatments were able to decrease disease incidence and lesion diameters. It was suggested that this effect resulted from the regulated ROS content (superoxide and hydrogen peroxide) and increased antioxidant activities (CAT and SOD). Similarly, Macarisin et al. [65] highlighted the ROS-mediated action of yeast antagonists *Metschnikowia fructicola* (strain 277) and *Candida oleophila* (strain 182) in postharvest treatment of citrus fruits and apples. The treatment of inoculating both yeasts onto citrus and apple wounds increased hydrogen peroxide content about fourfold after 18 h inoculation. Living *M. fructicola* cells were also found around the wound, indicating the yeast's ability to tolerate elevated ROS content. Thus, the use of some species of yeasts could be a solution to defeat fungi attack through the regulation of ROS content in the fruit cells.

Treatment of postharvest commodities with hormones has also shown some beneficial effects, which are understood to be the consequence of ROS regulation and increased antioxidant capacity. The use of acibenzolar-S-methyl (ASM), a functional analog of salicylic acid, decreased lesions caused by *Trichothecium roseum* in muskmelon (*Cucumis melo* L. cv. Yujingxiang). This effect was correlated with elevated concentrations of H_2_O_2_ and O_2_, increased activities of NADPH oxidase (NOX), SOD, and APX, and inhibition of CAT [66]. Further, salicylic acid treatment of cornelian cherry fruit (*Cornus mas*) significantly increased DPPH scavenging activity. This led to higher total phenols, flavonoids, anthocyanins, and ascorbic acid contents and enhanced PAL enzyme activity [67]. Senescence in cornelian cherry fruit was also delayed by the application of sodium nitroprusside (SNP) and *γ*-aminobutyric acid (GABA) in the postharvest cold storage condition. Both chemicals reduce H_2_O_2_ production, thus further ameliorating fruit browning. This outcome was also a consequence of improved activity of antioxidant enzymes, such as SOD, CAT, APX, and GR [68].

The application of animal analogous compounds has gained popularity in treating postharvest fruits. Once thought to contribute only to reducing oxidative stress in animals, sodium para-aminosalicylate is also able to inhibit ROS-mediated processes in postharvest litchi fruit. This tuberculosis drug delays pericarp ripening during senescence [6]. Similarly, melatonin, the famous sleep hormone in mammals, is capable of regulating ROS and delaying postharvest senescence in peach fruit [7]. Melatonin occurs widely and naturally in plants and can be extracted and upscaled for various applications.

Inorganic chemicals and moderate heat shock have also been described to extend shelf life of vegetables by modulating ROS. Biofortification of 15 mM potassium in KCl of tomato (*Solanum lycopersicum* L.) improved the quality during postharvest storage at 4°C [69] through limited loss of weight, water, and antioxidant content. Fumigation of fresh broccoli (*Brassica oleracea*) with hydrogen sulfide (H_2_S) was also able to improve activity of antioxidant enzymes, while reducing ROS production [70]. Prior to cold storage, heat shock treatment of spinach (*Spinacia oleracea*) at 40°C delayed leaf senescence and increased ascorbic acid content [71].

More sophisticated means of ROS postharvest management are genetic engineering and nanotechnology. In cassava (*Manihot esculenta* Crantz), the rapid postharvest physiological deterioration hurts farmers almost every harvest season. This problem is caused mainly by the accumulation of phenols and their oxidation which alters taste and instills brown color in the roots. Xu et al. [13] overproduced SOD and CAT by inserting an extra copy each of Me-*Cu/ZnSOD* and Me-*CAT1* into the cassava genome, which is controlled by a vascular-specific promoter p54. The transgenic plants showed a superior characteristic in terms of enzymatic activity of SOD and CAT. Further, reduced concentrations of malondialdehyde, lipid peroxidation, and H_2_O_2_ were observed in the transgenic line as compared to the wild variant. The use of nanocomposite packaging for postharvest enoki mushroom (*Flammulina velutipes*) has also significantly decreased ROS accumulation and lipid peroxidation activity. The nanocomposite packaging material contains 35% wt. Nano-TiO_2_ in the range of 40–60 nm [8] is well known for its photocatalytic capability in degrading ethylene, a fruit-ripening hormone [9].

## 7. Summary

The loss of crops primarily takes place in the postharvest process, especially in developing countries with limited access to high-quality technology. The loss not only affects farmers but also consumers, as low-quality fruits and vegetables entail reduced health benefits. The elevated ROS content in fresh commodities, a consequence of poor postharvest management, removes the advantage of a good antioxidant supply required to maintain a healthy body. For plants, ROS are not mainly deleterious as many are also involved in the signaling processes that determine crucial development stages. However, in most postharvest fruits and vegetables, no developmental stage is expected to occur and changes related to overripening or senescence should be prevented. Hence, the presence of ROS has to be balanced by antioxidant production. This concept is largely applied in developing methods to restrain microbial infection in postharvest commodities. Treatments are given to increase ROS content to a toxic level for microbes, which is then followed by antioxidant production to protect the plant cells. In this case, the fruits and vegetables are still acceptable for markets, but the antioxidant content might not be optimum for consumers. Hence, postharvest methods to maintain, if not increase, antioxidant content while preserving the physical appearance of fruit and vegetable commodities need to be developed. Genetic engineering might be a suitable solution, because overexpressing the antioxidant coding gene(s) is not necessarily coupled with ROS synthesis. The use of an inducible promoter will allow the harvested plant tissues to increase production of antioxidant content, preferably nonenzymatic, using spatiotemporal application of the inducer when the commodities are ready for sale. This approach is considered to reduce the chances of antioxidant degradation during postharvest and distribution.

## Figures and Tables

**Table 1:** Approaches of postharvest management to maintain quality, which are regulated by the balance of reactive oxygen species and antioxidant contents or activities.

Commodity	Treatment	Results	Reference
Water bamboo shoot (*Zizania aquatica* L.)	Modified atmosphere packaging and low temperature storage	Lowered production rate of superoxide and hydrogen peroxide	[62]
		Maintained high activities of SOD, CAT, and APX	

Loquat (*Eriobotrya japonica*)	Chitosan coating	Enhanced activity of SOD, CAT, and APX	[59]
		Inhibited activity of polyphenol oxidase (PPO) and guaiacol peroxidase (GPX)	

Strawberry (*Fragaria ananassa*)	Chitosan coating and cold storage	Delayed changes in the content of ascorbic acid, polyphenol, anthocyanin, flavonoid, and total antioxidant capacity	[63]
		Enhanced activity of CAT, APX, and lipoxygenase (LOX) activity	

Banana (*Musa acuminata*)	Coating with chitosan and putrescine	Increased phenolic compound contents and total antioxidant activity	[64]

Citrus (*Citrus sinensis* L.)	Antagonistic yeast (*Pichia membranaefacien*s)	Increased resistance to pathogenic fungi	[11]
		Regulated O_2_^−^ and H_2_O_2_	
		Enhanced activity of SOD and CAT	

Apple (*Malus domestica*) and citrus (*Citrus sinensis* L.)	Antagonistic yeasts (*Metschnikowia fructicola* and *Candida oleophila*)	Increased H_2_O_2_ content	[65]

Muskmelon fruit (*Cucumis melo* L.)	Acibenzolar-S-methyl (ASM), a functional analog of salicylic acid	Increased resistance to fungi	[66]
		Elevated concentration of O_2_^−^ and H_2_O_2_	
		Enhanced activity of NADPH oxidase (NOX), SOD, and APX	

Cornelian cherry fruit (*Cornus mas*)	Salicylic acid	Increased content of phenols, flavonoids, anthocyanins, and ascorbic acid	[67]
		Enhanced activity of DPPH scavenging and phenylalanine ammonia-lyase (PAL) enzyme	

Cornelian cherry fruit (*Cornus mas*)	Sodium nitroprusside (SNP) and g-aminobutyric acid (GABA) in cold storage	Reduced H_2_O_2_ production	[68]
		Enhanced activity of SOD, CAT, GR, and APX	

Litchi (*Litchi chinensis*)	Sodium para-aminosalicylate	Inhibited ROS production	[6]
		Enhanced activity of SOD, CAT, and glutathione peroxidase (GPX)	

Peach (*Prunus persica*)	Melatonin	Decreased content of O_2_^−^, H_2_O_2_, and malondialdehyde and activity of LPX	[7]
		Enhanced activity of SOD, CAT, peroxidase, and APX	

Tomato (*Solanum lycopersicum* L.)	Biofortification with potassium	Limited weight and water loss	[69]
		Improved concentration of lycopenes and flavonoids	

Broccoli (*Brassica oleracea*)	Fumigation with hydrogen sulfide	Decreased content of O_2_^−^, H_2_O_2_, and malondialdehyde	[70]
		Enhanced activity of GPX, APX, CAT, and GR and concentration of carotenoids, anthocyanins, and ascorbate	

Spinach (*Spinacia oleracea*)	Moderate heat shock	Enhanced chlorophyll content and potential quantum yield of PSII (Fv/Fm)	[71]
		Increased content of ascorbic acid and H_2_O_2_	

Cassava (*Manihot esculenta*)	Overexpression of SOD and CAT	Decreased activity of LPX and content of H_2_O_2_ and malondialdehyde	[13]

Shitake mushroom (*Flammulina velutipes*)	Nanocomposite packaging	Decreased content of O_2_^−^, H_2_O_2_, and malondialdehyde	[8]
